# Supplementary material for: CNPY3 Promotes Human Breast Cancer Progression and Metastasis via Modulation of the Tumor Microenvironment
Source: Curr Issues Mol Biol. 2025 Oct 24;47(11):883. doi: 10.3390/cimb47110883 (PMC12651230; doi:10.3390/cimb47110883)
Supplement: Supplementary file 1 [file cimb-47-00883-s001.zip › Supplemental Figures.pdf]

**Figure S1**

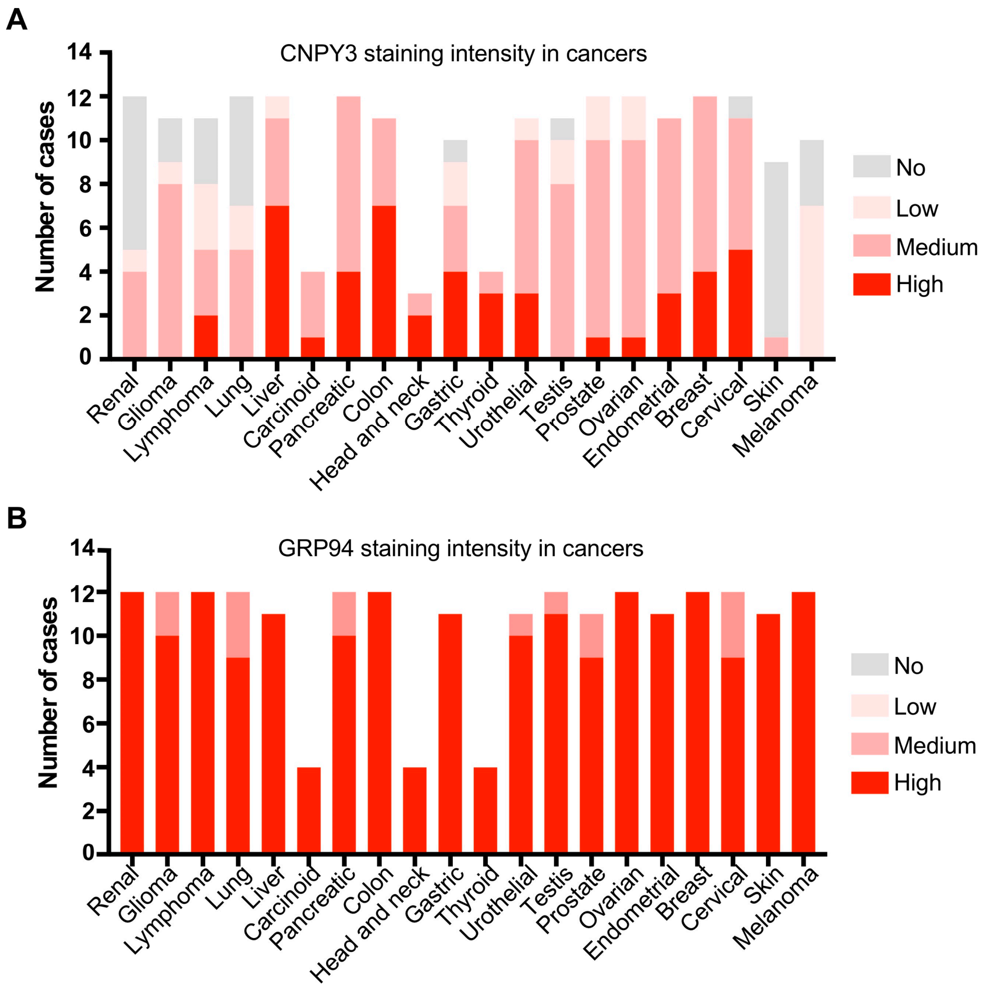


**Figure S1. CNPY3 and GRP94 protein expression in human cancers.** CNPY3 and GRP94 protein expression were investigated in 20 human cancers by Human protein atlas. **(A)** Moderate to strong CNPY3 cytoplasmic staining was found in most cancer tissues. **(B)** A majority of human cancers displayed strong cytoplasmic staining of GRP94.

**Figure S2**

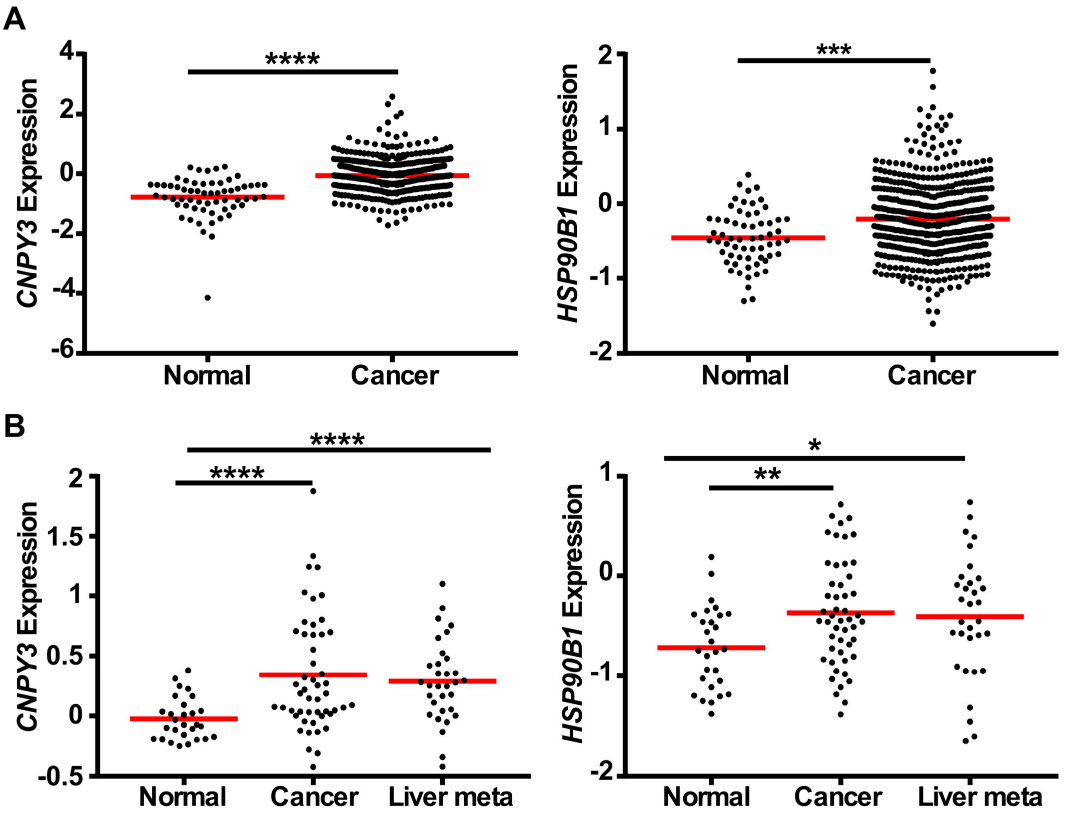


**Figure S2. High expression of *both CNPY3* and *HSP90B1* in human cancers. (A)** High mRNA levels of both *CNPY3* and *HSP90B1* in breast cancers by analyzing TCGA breast cancer dataset (n=593). **(B)** High mRNA levels of both *CNPY3* and *HSP90B1* in colon cancers and liver metastatic colon cancers by analyzing Ki colon dataset (n=123). **P*<0.05, ***P*<0.01, ****P*<0.001, *****P*<0.0001.
