# Supplementary material for: CNPY3 Promotes Human Breast Cancer Progression and Metastasis via Modulation of the Tumor Microenvironment
Source: Curr Issues Mol Biol. 2025 Oct 24;47(11):883. doi: 10.3390/cimb47110883 (PMC12651230; doi:10.3390/cimb47110883)
Supplement: Supplementary file 1 [file cimb-47-00883-s001.zip › Supplemental Table S1.pdf]

Table S1. The dataset information presented in present study for breast and colorectal cancer

Dataset name	PMID	Description, cases	Array type	Experiment type
**Breast cancer**				
Curtis breast dataset	22522925	1992 breast carcinoma,<br>144 paired normal breast samples	Illumina Human HT-12 V3.0 R2 Array<br>Measured 19,273 genes, 48,803 reporters	mRNA
Zhao breast dataset	15034139	61 breast carcinomas<br>3 normal breast samples	Platform not pre-defined in Oncomine<br>Measured 12,482 genes, 27,276 reporters	mRNA
Richardson breast dataset	16473279	40 ductal breast carcinomas<br>7 normal breast samples	Human Genome U133 Plus 2.0 Array<br>Measured 19,574 genes, 54,675 reporters.	mRNA
TCGA breast dataset	TCGA data	532 invasive breast carcinomas,<br>61 paired normal breast, 3 paired metastatic samples	Platform not pre-defined in Oncomine<br>Measured 20,423 genes, 111,123 reporters	mRNA
Perou breast dataset	10963602	62 breast carcinomas<br>3 normal breast samples	Platform not pre-defined in Oncomine<br>Measured 6,625 genes, 8,838 reporters	mRNA
Sorlie breast dataset	11553815	78 breast carcinomas<br>4 normal breast samples	Platform not pre-defined in Oncomine<br>Measured 6,304 genes, 7,937 reporters	mRNA
Sorlie breast 2 dataset	12829800	160 breast carcinomas<br>4 normal breast samples	Platform not pre-defined in Oncomine<br>Measured 6,197 genes, 7,705 reporters.	mRNA
Gluck breast dataset	21373875	154 invasive breast carcinomas<br>4 normal breast samples	Platform not pre-defined in Oncomine<br>Measured 17,862 genes, 41,788 reporters.	mRNA
Lu breast dataset	18297396	129 breast carcinoma samples	Human Genome U133 Plus 2.0 Array<br>Measured 19,574 genes, 54,675 reporters.	mRNA
Kao breast dataset	21501481	327 breast carcinoma samples	Human Genome U133 Plus 2.0 Array<br>Measured 19,574 genes, 54,675 reporters	mRNA
Miyake breast dataset	22320227	115 breast carcinoma samples	Human Genome U133 Plus 2.0 Array<br>Measured 19,574 genes, 54,675 reporters	mRNA
Schmidt breast dataset	18593943	200 invasive breast carcinoma samples	Human Genome U133A Array<br>Measured 12,624 genes, 22,283 reporters	mRNA
Hatzis breast dataset	21558518	508 invasive breast carcinoma samples	Human Genome U133A Array<br>Measured 12,624 genes, 22,283 reporters	mRNA
Wang breast dataset	15721472	286 breast carcinoma samples	Human Genome U133A Array<br>Measured 12,624 genes, 22,283 reporters	mRNA
Ivshina breast dataset	17079448	289 breast carcinoma samples	Human Genome U133A Array<br>Measured 12,624 genes, 22,283 reporters	mRNA
Boersma breast dataset	17999412	95 breast carcinoma samples	Human Genome U133A Array<br>Measured 12,624 genes, 22,283 reporters.	mRNA
Finak breast dataset	18438415	53 breast tumor stroma samples<br>6 normal breast stroma samples	Agilent Human Genome 44K<br>Measured 19,189 genes, 41,000 reporters	mRNA
Symmans breast dataset	20697068	195 invasive breast carcinoma samples	Human Genome U133A Array<br>Measured 12,624 genes, 22,283 reporters	mRNA
Minn breast dataset	16049480	99 breast cancer samples	Human Genome U133A Array<br>Measured 12,624 genes, 22,283 reporters	mRNA
Bittner breast dataset	No	336 breast carcinoma samples	Human Genome U133 Plus 2.0 Array<br>Measured 19,574 genes, 54,675 reporters	mRNA
Desmedt breast dataset	17545524	198 breast cancer samples	Human Genome U133A Array<br>Measured 12,624 genes, 22,283 reporters	mRNA

Esserman breast dataset	22198468	129 breast carcinoma samples	Agilent Human Genome 44K<br>Measured 19,189 genes, 41,000 reporters	mRNA
Bonnefoi breast dataset	18024211	160 breast carcinoma samples	Affymetrix Human X3P Array<br>Measured 19,139 genes, 61,348 reporters	mRNA
Julka breast dataset	18382427	44 breast carcinoma samples	Agilent Human 1A Oligo Microarray v2<br>Measured 16,724 genes, 21,073 reporters	mRNA
Chang breast dataset	12907009	24 invasive breast carcinoma samples	Human Genome U95A-Av2 Array<br>Measured 8,603 genes, 12,651 reporters	mRNA
Stickeler breast dataset	21769435	57 breast carcinoma samples	Agilent Human Genome 44K<br>Measured 19,189 genes, 41,000 reporters	mRNA
Ma breast 4 datasets	19187537	38 ductal breast carcinomas<br>28 normal breast samples	Affymetrix Human X3P Array<br>Measured 19,139 genes, 61,348 reporters.	mRNA
Vandevijver breast dataset	12490681	295 breast carcinoma samples	Platform not pre-defined in Oncomine<br>Measured 14,719 genes, 23,130 reporters	mRNA
Weigelt breast dataset	14665696	16 breast carcinoma samples	Platform not pre-defined in Oncomine<br>Measured 10,335 genes, 18,102 reporters	mRNA
Schuetz breast dataset	16707453	14 breast cancer samples	Human Genome U133 Plus 2.0 Array<br>Measured 19,574 genes, 54,675 reporters	mRNA
**Colon dataset**				
Ki colon dataset	17640062	103 matched samples, normal colon, colon cancer normal liver and liver metastasis	Platform not pre-defined in Oncomine<br>Measured 9,256 genes, 15,783 reporters	mRNA
TCGA colon dataset	TCGA data	215 colorectal adenocarcinomas<br>22 paired normal colorectal samples	Platform not pre-defined in Oncomine<br>Measured 20,423 genes, 111,123 reporters	mRNA
Kaiser colon dataset	17615082	100 colorectal carcinomas<br>5 normal colon samples	Human Genome U133 Plus 2.0 Array<br>Measured 19,574 genes, 54,675 reporters	mRNA
Hong colon dataset	20143136	70 colorectal carcinomas<br>12 normal colon samples	Human Genome U133 Plus 2.0 Array<br>Measured 19,574 genes, 54,675 reporters	mRNA
Staub colon datasete	19399471	62 colorectal carcinoma samples	Human Genome U133A Array<br>Measured 12,624 genes, 22,283 reporters	mRNA
Smith colon 2 datasets	19914252	55 colorectal adenocarcinoma samples	Human Genome U133 Plus 2.0 Array<br>Measured 19,574 genes, 54,675 reporters	mRNA
Graudens colon dataset	16542501	48 colorectal carcinomas with 30 liver metastases<br>12 normal colon samples	Platform not pre-defined in Oncomine<br>Measured 6,248 genes, 18,976 reporters	mRNA
